# Pathogenomic Analysis of a Novel Extensively Drug-Resistant *Citrobacter freundii* Isolate Carrying a bla_NDM-1_ Carbapenemase in South Africa

**DOI:** 10.3390/pathogens9020089

**Published:** 2020-01-31

**Authors:** Yogandree Ramsamy, Koleka P. Mlisana, Daniel G. Amoako, Mushal Allam, Arshad Ismail, Ravesh Singh, Akebe Luther King Abia, Sabiha Y. Essack

**Affiliations:** 1 Medical Microbiology, College of Health Sciences, University of KwaZulu-Natal, Durban 4000, South Africa; Singhra@ukzn.ac.za; 2 National Health Laboratory Services, Durban 4000, South Africa; MlisanaK@ukzn.ac.za; 3 Antimicrobial Research Unit, College of Health Sciences, University of KwaZulu-Natal, Durban 4000, South Africa; lutherkinga@yahoo.fr (A.L.K.A.); essacks@ukzn.ac.za (S.Y.E.); 4 Infection Genomics and Applied Bioinformatics Division, Antimicrobial Research Unit, College of Health Sciences, University of KwaZulu-Natal, Durban 4000, South Africa; amoakodg@gmail.com; 5 Sequencing Core Facility, National Institute for Communicable Diseases, National Health Laboratory Service, Johannesburg 2131, South Africa; MushalA@nicd.ac.za (M.A.); ArshadI@nicd.ac.za (A.I.)

**Keywords:** Pathogenomics, Novel, *Citrobacter freundii*, bla_NDM-1_, Mobilome, South Africa, Carbapenemase

## Abstract

Pathogenomic analysis was performed on a novel carbapenem-resistant *Citrobacter freundii* isolate (H2730R) from a rectal swab of an adult male patient admitted to a tertiary hospital, Durban, South Africa. H2730R was identified using selective media and API 20e kit. Confirmatory identification and antibiotic susceptibility testing were performed using the VITEK II. H2730R was whole-genome sequenced on the Illumina MiSeq platform. H2730R was resistant to all tested antibiotics except tigecycline and was defined as ST498 by the *C. freundii* multilocus sequence typing (MLST) database. The estimated pathogenic potential predicted a higher probability (P_score_ ≈ 0.875), supporting H2730R as a human pathogen. H2730R harbored 25 putative acquired resistance genes, 4 plasmid replicons, 4 intact prophages, a class 1 integron (IntI1), 2 predominant insertion sequences (IS3 and IS5), numerous efflux genes, and virulome. BLASTn analysis of the bla_NDM-1_ encoding contig (00022) and its flanking sequences revealed the bla_NDM-1_ was located on a plasmid similar to the multireplicon p18-43_01 plasmid reported for the spread of carbapenem resistance in South Africa. Phylogenomic analysis showed clustering of H2730R with CF003/CF004 strains in the same clade, suggesting a possible association between *C. freundii* strains/clones. Acquiring the p18-43_01 plasmid containing bla_NDM-1_, the diversity, and complex resistome, virulome, and mobilome of this pathogen makes its incidence very worrying regarding mobilized resistance. This study presents the background genomic information for future surveillance and tracking of the spread of carbapenem-resistant *Enterobacteriaceae* in South Africa.

## 1. Introduction

*Citrobacter freundii (C. freundii)* is a rod-shaped, Gram-negative, aerobic member of the Enterobacterales family that is an intestinal commensal in humans and animals [1,2]. It is a known cause of nosocomial infections associated with the biliary tract, respiratory tract, urinary system, and central nervous system, including meningitis, neonatal sepsis, and infectious diarrhea [3,4]. *C. freundii* can persist in their hosts for a long time and acquire resistance mechanisms, making the treatment of their infections challenging [4]. The emergence and dissemination of drug-resistant *C. freundii* in humans, animals, and the environment have previously been described, making this pathogen a potential reservoir for the spread of antimicrobial-resistant genes [1,2,4,5,6,7].

Carbapenem resistance is a growing concern in *C. freundii,* as carbapenems are regarded as antimicrobial agents of last resort used to treat life-threatening infections caused by these pathogens [8]. The easy mobilization of carbapenemase encoding genes, due to their locations on mobile genetic elements (MGEs), has favored the acquisition of carbapenem resistance by diverse *Enterobacterales* [9]. These MGEs, specifically plasmids, prophages, insertion sequences (IS), and integrons, are known to influence microbial virulence and pathogenicity via horizontal gene transfer (HGT) [10,11]. HGT allows new genomic traits to be acquired from other unrelated bacteria and remains the most effective means of bacterial evolution, causing the dissemination of antibiotic resistance and associated disease pathogenesis [12,13]. In this study, we present the emergence of a novel carbapenem-resistant sequence type (ST498) of *C. freundii* isolated from a rectal swab of an adult male patient admitted to a tertiary healthcare facility in Durban, South Africa, harboring a diverse resistome, virulome, and mobilome. This investigation forms part of a broader study specifically designed to isolate carbapenemase-producing *Enterobacteriaceae* (CPE) for patients at admission in a public hospital in the province. We also show the phylogenomic relationship between this novel isolate (H2730R) and all the deposited *C. freundii* genomes from South Africa at the Pathosystems Resource Integration Center (PATRIC) online platform. 

## 2. Results

### 2.1. Isolation, Phenotypic Confirmation of Carbapenem Resistance, and Antibiotic Susceptibility Testing (AST) 

The carbapenem-resistant *C. freundii* strain (H2730R) was isolated using the selective media and confirmed using the VITEK II AST-N255 automated platform. Antibiotic susceptibility testing (AST) revealed that H2730R was extensively drug-resistant (XDR) (Table 1).

### 2.2. General Genomic Features of H2730R

**Figure A1:**
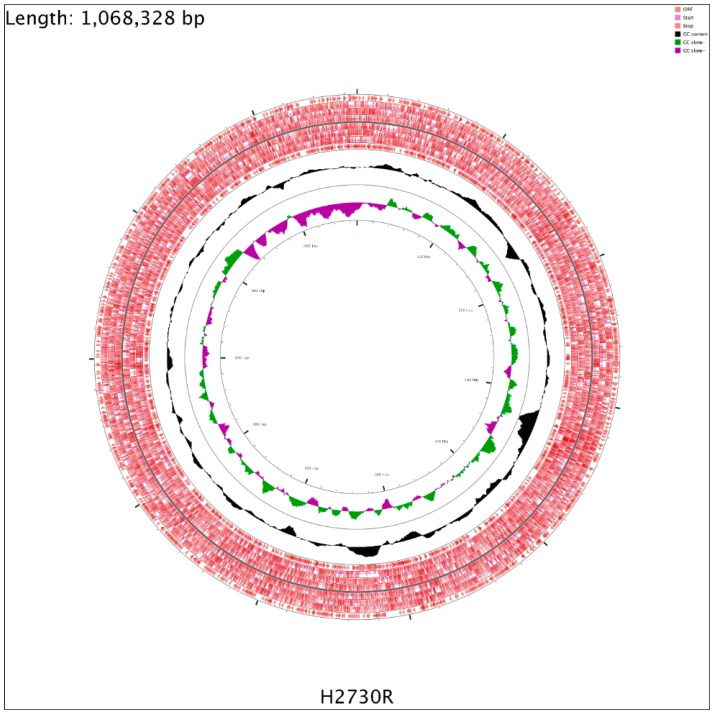
Graphic depiction of the H2730R genome belonging to ST498. The two outer circles show the open reading frame (ORF–in mauve). The inner circle shows the GC skew, with green and purple indicating positive and negative values, respectively. The GC content is indicated in black. This genome map was visualized using the CGView Server.

The CG Viewer Server was used for the genomic visualization of the isolate (Figure A1; Appendix A). The *C. freundii* (H2730R) genomic features are shown in Table A1. The size, GC content, number of contigs, N50, and L50 of the H2730R genome were 5.29 Mbp, 51.80%, 58, 518368, and 4, respectively. Annotation with RAST and PGAP resulted in 5006 protein-coding genes, 70 RNAs, and 12 tRNAs (Table A1). The protein-coding genes (CDSs) and non-protein-coding genes were assigned to 27 clusters of orthologous groups (COG) and functional categories (Table A1). 

### 2.3. WGS-Based Confirmation and Multilocus Sequence Typing (MLST)

The identification of H2730R isolate was confirmed with generated genomic data via the Global Platform for Genomic Surveillance (Pathogenwatch). In silico determination of the classical sequence type from WGS data using the *C. freundii* MLST scheme resulted in a previously undescribed MLST comprising a new allelic combination for *arca_5, aspc_16, clpx_14, dnag_54, fadd_103, lysp_5, mdh_15*. Allele sequences were submitted for curation and assigned to the new ST 498 by the *C. freundii* PubMLST database. BURST (Based Upon Related Sequence Types) analysis identified the novel ST498 as a satellite clone (more distantly related strain) with no single-locus variant (SLV) or double-locus variant (DLV) of globally curated *C. freundii* STs. Of note, the new ST had different allelic profiles from the five deposited *C. freundii* genomes from South Africa.

### 2.4. WGS Analysis of Resistance Genes and Genetic Support

WGS analysis via the different databases revealed 25 putative acquired antibiotic resistance genes responsible for resistance to the various antibiotics found in the isolate, which corroborated the phenotypic profile (Table 2). Chromosomal mutation in GyrA [S83I] and the plasmid-mediated quinolone resistance genes (*aac (6')-Ib-cr and qnrB1*) were found. Most of the resistance genes were plasmid-borne (Table 2). Resistance to carbapenems was linked to the New Delhi metallo-β-lactamase-1 (*bla*_NDM-1_) belonging to the sub-class B1. The BLASTn analysis of the bla_NDM-1_ carbapenemase containing contig (00022) and its flanking sequences matched the 212.3 Kbp multireplicon plasmid p18-43_01 (GenBank accession number **CP023554.1**). The PlasmidFinder v1.3 identified four plasmid replicons (Inc A/C2, Inc FIB(pB171), Inc FII(Yp), and Inc Q1). 

**Figure 1:**
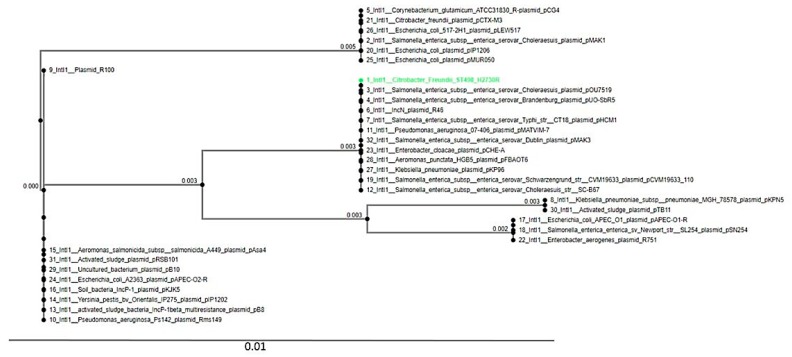
A phylogenetic tree showing the association between Integron_Integrase_IntI1 of *C. freundii* H2730R and other predicted closely related *IntI1* integrases in diverse species.

The PHAge Search Tool (PHAST) detected four intact phages (Escher_HK639, Entero_c_1, Salmon_RE_2010, and Salmon_SJ46). The insertion sequences in the genomes belonging to the IS3 family (IS2) and IS5 family (IS903) were predicted by BLAST searches against contigs on the ISFinder database. In silico analysis identified the class 1 integron-integrase gene (IntI1) in H2730R. The relationship between the IntI1 integrase in H2730R and other closely related IntI1 integrases in diverse species obtained from the RAST Server was shown as a phylogenetic tree using the MAFFT online tool (Figure 1). The Intl1 of the H2730R strain had the closest similarity with the *Salmonella enterica subsp. enterica serovar Choleraesuis* plasmid pOu7519, as depicted in Figure 1.

Furthermore, 10 efflux pump systems known to be involved in drug resistance were identified (Table 2). They belonged to three main efflux systems, namely: ABC, ATP-binding cassette; MFS, major facilitator superfamily; and RND, resistance–nodulation–division (Table 2).

### 2.5. Pathogenicity, Defence Systems Mechanisms, and Virulome Predictions

H2730R had a 0.875 probability of being pathogenic to humans and was found to match with 110 pathogenic families. All the 110 pathogenic families were linked to members of the Enterobacterales, of which *Citrobacter koseri* ATCC BAA-895 was the organism with the highest possible pathogen linkage. The CRISPRCasFinder predicted two clustered, regularly interspaced, short palindromic repeat (CRISPR) arrays with no Cas systems. Of note, the in silico analysis revealed a Type II restriction-modification (R-M) defense system consisting of *Eco128I* (restriction enzyme) and *M. EcoRII* (methyltransferase)]. The whole-genome virulome analysis predicted virulence-encoded pathogenesis-associated proteins predominantly made up of adherence determinants, regulation, toxin, motility, antiphagoctyosis, invasion, and biofilm formation (Table 3).

### 2.6. Phylogenomic Analysis and Metadata Insights of Reported Citrobacter Freundii Isolates from South Africa

**Figure 2:**
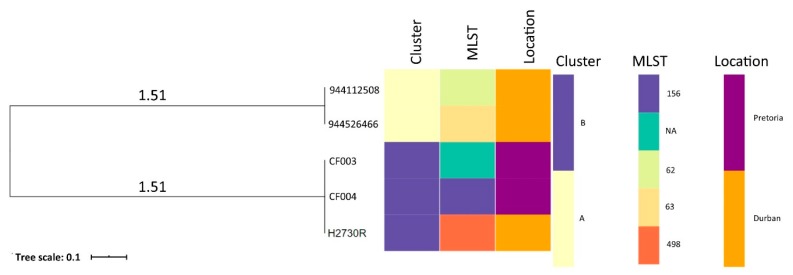
The whole-genome phylogenomic branch and metadata [MLST and Demographics (Location and Host)] coupled using Phandango showing the relationship between H2730R and all deposited *Citrobacter freundii* strains (n = 4) from South Africa. All the isolates were from a human host.

The phylogenetic relationship and epidemiological distribution of all deposited *C. freundii* genomes on GenBank from South Africa are depicted in Figure 2 in which isolates of the same cluster are highlighted in the same color. The tree analysis, coupled with metadata, revealed two clusters (A and B) of *C. freundii* with insights into the diversity of clones in South Africa (Table A2). Of note, the H2730R was related to the CF003 and CF004 strains (Cluster B). All the isolates were from human hosts.

## 3. Discussion

Carbapenem resistance in Enterobacteriaceae has become a major public health threat that requires urgent attention. In this study, we use whole-genome sequencing (WGS) and bioinformatics analysis to describe the emergence of a novel carbapenem-resistant sequence type (ST498, satellite clone) of *C. freundii* isolated from the rectal swab of an adult male patient admitted to a tertiary healthcare facility in Durban, South Africa, harboring a diverse resistome, virulome, and mobilome. 

H2730R was categorized as XDR due to its phenotypic resistance to all tested antibiotics except tigecycline [14] (Table 1). The carbapenem phenotypes of the strain, as identified on selective chromogenic screening medium, were confirmed by the presence of carbapenemase (bla_NDM-1_) (Table 1 and Table 2). The 100% concordance between phenotypes and predicted resistome annotation data reiterated the reliability of WGS for the accurate prediction of antibiotic resistance [15,16]. The *C. freundii* H2730R genome harbored a total of 25 putative resistance genes, which were predominantly plasmid-borne (Table 2), indicating an acquisition and the potential for spread via horizontal resistome transfer between H2730R and other species. Resistance to ciprofloxacin was mediated by GyrA [S83I] chromosomal mutation, plasmid-mediated quinolone resistance genes (PMQR) (*aac(6')-Ib-cr and qnrB1*), and efflux genes (Table 2) [17]. Sekyere and Amoako et al. (2017) [15] reported that high-level fluroquinolone resistance was mediated by efflux, PMQR genes, and gyrA mutations in Enterobacterales, including *Citrobacter freundii*, which supported our results. More so, efflux genes belonging to three main efflux pump systems (Table 2) reported to play vital roles in antibiotic resistance in Enterobacterales were also identified [18,19]. However, the mere presence of these efflux genes does not directly implicate resistance, and hence further studies on their expression levels will be needed to ascertain the possibility of efflux pump hyperexpression [18]. The diverse resistance mechanisms (plasmid-borne, chromosomal, and efflux) involved in H2730R resistance indicate the various processes the organism expresses to survive the effects of antibiotics and other biocides (Table 2). 

The National Center for Biotechnology Information (NCBI) microbial nucleotide BLAST search of the bla_NDM-1_ carbapenemase containing contig (00022) and its flanking sequences in the isolate revealed the bla_NDM-1_ was located on a plasmid similar to the 212.3 Kbp multireplicon p18-43_01 plasmid (accession no. **CP023554.1**) reported for the spread of carbapenemases in Enterobacterales (including *K. pneumoniae* [n = 4], *Citrobacter freundii* [n = 1], *Serratia marcescens* [n = 11], and *Enterobacter* family complex [n = 7]) in the private [20] and public healthcare sector [21] in Durban, South Africa. This implicates a possible bla_NDM-1_ acquisition as well as nosocomial spread and development of an XDR genetic lineage via this local plasmid across sectors in the KZN province, which is very worrisome. The four plasmid replicon genes (Inc A/C2, Inc FIB(pB171), Inc FII(Yp), and Inc Q1) identified via the PlasmidFinder supported the multireplicon plasmid nature of the p18-43_01. This global spread of carbapenem resistance through the acquisition of plasmid encoding carbapenemase has been highly documented in the literature [22,23,24]. 

Moreover, H2730R contained two insertion sequences (IS3 family and IS5 family) whose genetic transposition could help in the plasticity and concomitant adaptability of H2730R phenotypic traits, including catabolism, pathogenicity, resistance to antibacterial agents, and virulence [25]. The IS2 (IS3 family) has been reported to cause erythromycin and fluoroquinolone resistance through the formation of hybrid promoters [25]. Additionally, the IS930 (IS5 family) has been implicated in carbapenem and metal ion (silver) resistance in *Enterobacter aerogenes* [26] and *E. coli*, respectively. Further analysis of H2730R revealed prophage with possible origination from diverse sources (*E. coli* and *Salmonella*) [27]. For instance, the two prophages Salmon_RE_2010 and Salmon_SJ46 were initially identified in *Salmonella enterica subsp. enterica serovar Enteritidis* and Salmonella *enterica subsp. enterica serovar Indiana,* respectively [28]. This supports a possible exchange of genetic traits via horizontal transfer between Enterobacterales. Class 1 integrons, reported to be associated with multiple classes of antibiotic resistome, including β-lactams, quinolones, and aminoglycosides worldwide, were found [29]. However, the full mobile gene cassettes and integron genetic structure could not be determined as they were truncated into different contigs during the assembly process. 

The pathogenic potential (P_score_) is a machine learning algorithm used for the in silico prediction of the possibility of a strain being a human pathogen with the probability ranging from 0 to 1. This estimation of the pathogenic potential using trained algorithms to differentiate between pathogenic or commensal strains predicted a higher probability (P_score_ ≈0.875) suggesting H2730R as a potential human pathogen. Further, in silico analysis of H2730R predicted the presence of two CRISPR arrays and a Type II restriction–modification (R-M) defense system which offers bacterial protection against viral attacks and thus increases its survival and adaptability in the microbial landscape [30,31]. 

Moreover, the genomic virulome analysis revealed a battery of different virulence encoded proteins from diverse sources. These virulence determinants were predominantly made up of adherence determinants which aid in the complement-independent attachment to host mammalian cells [32]. H2730R possessed toxins to induce cell membrane damage, trigger cytokine formation, and reduce or kill neutrophils [33]. Interestingly, the strain also harbored the *ibeB* and *pgaC* genes, which contribute to invasion, biofilm formation, and adhesion to eukaryotic host cells. Additionally, H2730R harbored the two-component regulation system (*PhoPQ*) activated by low divalent cations magnesium (Mg^2+^) and calcium (Ca^2+^) levels and required for intracellular survival, cationic antimicrobial peptides (CAMPs) resistance, and stimulation of cytokine secretion [34]. The higher virulence factor homology existing between H2730R and other Gram-negative species (Table 3) suggests that they were possibly obtained horizontally, necessitating further studies on transconjugation and/or transcriptomics to ascertain this assertion. The detection of the virulome can inform vaccine development by delineating possible bacterial protein targets. 

Phylogenomic analysis, coupled with metadata, showed clustering of H2730R (Durban) with CF003/CF004 strains in the same cluster (B) suggesting a possible association of *C. freundii* strains/clones. However, not much inference could be drawn from the tree analysis due to the small number of deposited *C. freundii* genomes from South Africa. It is thus recommended that more studies be carried out in all the sectors (human, animal, and environment) to monitor the emergence and spread of XDR *C. freundii* using high throughput technologies such as whole-genome sequencing (WGS). Moreover, there was limited data on the clinical information, history and/or clinical examination of the patient, and all deposited *C. freundii* genomes obtained from the human host, which could be very useful in comparative phylogenomic analysis. Ultimately, a combination of demographics, clinical information, and WGS data coupled with graphical analysis should be applied to offer insights into the spread of pathogens and increase confidence during molecular epidemiological investigations [35].

## 4. Materials and Methods 

### 4.1. Ethical Approval

This study was approved by the Biomedical Research Ethics Committee (approval no: **BE599/16**, a substudy of **BCA444/16**), College of Health Sciences, University of KwaZulu-Natal (UKZN).

### 4.2. Identification of the Isolate

Selective chromogenic screening medium, CHROMID^®^ CARBA (BioMérieux, Marcy l'Étoile, France), was used for the isolation of carbapenemase-producing Enterobacteriaceae from a screening rectal swab obtained from an adult patient. The CHROMID^®^ CARBA (BioMérieux, Marcy l'Étoile, France) agar plate was inoculated with the following control strains: carbapenemase-negative *K. pneumoniae* ATCC 700603 and carbapenemase-positive *K. pneumoniae* ATCC BAA-1705.

### 4.3. Antibiotic Susceptibility Testing (AST)

Confirmatory phenotypic microbial identification and antibiotic susceptibility testing (AST) was performed on the VITEK II AST-N255 automated platform (BioMérieux, Marcy l'Étoile, France). The universal antimicrobial test panel included: penicillin, ampicillin, amoxicillin-clavulanate, ceftriaxone, cefepime, cefuroxime, cefoxitin, ceftazidime, imipenem, meropenem, ertapenem, piperacillin-tazobactam, amikacin, gentamicin, nitrofurantoin, trimethoprim/sulfamethoxazole, ciprofloxacin, and tigecycline. The isolate was characterized as susceptible or resistant using CLSI-approved breakpoints [36].

### 4.4. DNA Purification, Genome Sequencing, and Preprocessing of Sequence Data

The isolate was streaked onto a nutrient agar (NA) (Sigma-Aldrich, St. Louis, USA) plate and incubated at 37 °C for 24 h. Following incubation, genomic DNA was extracted from a visibly pure culture using the Quick-DNA™ Fungal/Bacterial Miniprep Kit (Zymogen Research, USA, cat. no. D6005), quantified by Nanodrop 8000 (Thermo Scientific, Waltham, MA) and Qubit, and verified by agarose gel electrophoresis. A paired-end library (2 × 300 bp) was prepared using Illumina Nextera XT DNA Sample Preparation Kit and sequenced on a MiSeq machine (Illumina, USA). The generated sequenced reads were quality assessed and trimmed using the Skesa assembler (version 2.3). Default parameters were used for all software unless otherwise specified. The CheckM tool [37] was used to verify that the sequence reads were not from mixed species using lineage-specific marker sets from other genetically well-characterized, closely related *C. freundii* strains. 

### 4.5. Bioinformatic Analysis

#### 4.5.1. Genome Visualization and Annotation 

The raw reads were de novo assembled using the Skesa (version 2.3) assembler [38]. The genomes of the strains were visualized using the CG Viewer Server (Figure A1; Appendix A). The National Center for Biotechnology Information (NCBI) Prokaryotic Genome Annotation Pipeline (PGAP; version 4.3) [39] and Rapid Annotation using Subsystem Technology (RAST) Server (version 2.0) [40] were used for annotation of the size, GC content, number of contigs, N50, L50, average coverage, number of RNAs, and protein-coding sequences of the isolate. 

#### 4.5.2. WGS-Based Confirmation and Molecular Typing 

The generated contigs from the WGS data were used to confirm the identity of the isolate on the pathogen watch platform [41]. The assembled genome was submitted to the *C. freundii* MLST database, which predicted the allelic profiles of the seven housekeeping genes to assign the new sequence type [42]. An eBURST [43] algorithmic analysis was performed in the MLST database (https://pubmlst.org/cfreundii/) to ascertain whether the novel sequence type (ST) was a single-locus variant (SLV) or double-locus variant (DLV) or satellite (SAT, more distantly related strain) of known STs. The allelic profiles and STs of all deposited *C. freundii* genomes from South Africa in the PATRIC database were also predicted using the MLST database.

#### 4.5.3. Genomic Identification of the Antibiotic Resistome and Mobile Genetic Elements (MGEs)

The bacterial analysis pipeline of GoSeqIt via ResFinder [44], Antibiotic Resistance Gene-Annotation database (ARG-ANNOT) [45], and the comprehensive antibiotic resistance database (CARD) [46] tools were also used to annotate and identify antibiotic resistance genes. To determine if the resistance genes and their associated MGEs were plasmid-borne or chromosomal, the contigs bearing these resistance genes were BLASTed on BLASTn to determine if the closest nucleotide homologies were chromosomal or plasmids. Plasmid replicons were predicted through PlasmidFinder [47]. The PHAge Search Tool [48] server was used for the identification, annotation, and visualization of prophage sequences. Phage regions were extracted and analyzed in the NCBI nucleotide BLASTn service to confirm their identity. Insertion sequences and transposase in the genomes were predicted by blasting contigs on the ISFinder database [49] to find the common insertion sequences. Integrons in the genomes predicted by PGAP and RAST subsystems were blasted on the INTEGRALL database to find the actual integrons. Additionally, using the nucleotide sequences, the MAFFT multiple alignment program [50] was used to show the phylogenetic relationship between predicted and other closely related integrases in diverse species obtained from the RAST Server.

#### 4.5.4. Pathogenicity, Defence Systems, and Virulome Predictions

PathogenFinder [51] was used to predict pathogenicity towards human hosts. The CRISPRCasFinder was used to identify the putative CRISPR loci and Cas cluster in the draft genomes [52]. Restriction Modification Finder predicted the R-M system in the isolate [53]. Virulence determinants (sequences and functions) corresponding to different major bacterial virulence factors associated with *C. freundii* were searched for virulence factors with the pathogenic bacteria database, VFanalyzer [54], and validated by blasting assembled genomes to a pseudomolecule generated by concatenating a set of target genes using the NCBI in-house BLASTN tool.

### 4.6. Phylogenomic Analyses of C. freundii Isolates from South Africa 

The genome sequences of all five available genomes were downloaded from GenBank and PATRIC for genomic comparison and phylogenomic analysis via CSI Phylogeny-1.4 [55], an online service which identifies SNPs from WGS data, filters and validates the SNP positions, and then infers phylogeny based on concatenated SNP profiles [55]. A bootstrapped indicator with 100 replicates was applied to identify recombined regions and provide the phylogenetic accuracy in groups with little homoplasy. Figtree was used to edit and visualize the phylogenetic tree. The phylogeny was visualized alongside annotations for isolate demographics and WGS in silico molecular typing metadata using Phandango [56]. 

### 4.7. Data Availability

This whole-genome shotgun project has been deposited in DDBJ/ENA/GenBank under the accession no. VWTQ00000000. The version described in this paper is version VWTQ01000000. 

## 5. Conclusions

This study reports the comprehensive pathogenomics of an XDR *C. freundii* strain isolated from South Africa. The multireplicon plasmid p18-43_01 encoding the bla_NDM-1_ reported for carbapenem resistance coupled with the diversity of the resistome, virulome, and mobilome of this novel pathogen makes this incidence very worrying. This necessitates continuous resistance surveillance programs, including rectal screening, stringent infection control measures, and antibiotic stewardship policies to curb further emergence and spread of these antibiotic-resistant bacteria.

## Figures and Tables

**Table 1:** Susceptibility Pattern of Isolate H2730R.

Antibiotic	AST Profile	Minimum Inhibitory Concentrations (mg/L)
Amoxicillin-clavulanic acid	R	(32/16)
Piperacillin-tazobactam	R	(128/4)
Cefuroxime	R	(32)
Cefotaxime	R	(4)
Ceftriaxone	R	(4)
Ceftazidime	R	(16)
Cefepime	R	(16)
Cefoxitin	R	(32)
Imipenem	R	(4)
Meropenem	R	(4)
Ciprofloxacin	R	(1)
Gentamicin	R	(16)
Amikacin	R	(64)
Nitrofurantoin	R	(128)
Trimethoprim-sulfamethoxazole	R	(4/76)
Tigecycline	S	(2*)

S, susceptible; I, intermediate; R, resistant. * CLSI 2017 used for interpretation of resistance breakpoints for all antibiotics except that of Tigecycline where EUCAST resistance breakpoints (v 7.1) were used for interpretation.

**Table 2:** Resistance gene profile and efflux pump systems of Isolate H2730R.

Antibiotic class	Genes	Genomic location
β-lactams	*blaNDM-1, blaCMY-48, blaCTX-M-15, blaOXA-10, OXA-1, blaTEM-1B*	Plasmid
Aminoglycosides	*aph(3')-Ia, aac(6')-Ib-cr, aac(3)-Iia, aph(6)-Id, aadA1, aac(3)-Iid, rmtC, aph(3'')-Ib*	Plasmid
Fluroquinolones	*aac(6')-Ib-cr, qnrB1, GyrA(S83I)*	Plasmid and Chromosome (G*yrA*)
Fosfomycin	*fosA*	Chromosome
Trimethoprim	*dfrA23, dfrA7, dfrA14*	Plasmid
Rifampicin	*ARR-2*	Plasmid
Phenicols	*catB3, cmlA1*	Plasmid
Sulphonamides	*sul2, sul1*	Plasmid
Tetracycline	*tet(A)*	Plasmid
**Efflux pump systems**		
ATP-binding cassette (ABC)	*msbA*	Chromosome
Major facilitator superfamily (MFS)	*mdfA, mdtG*	Chromosome
Resistance– nodulation–division (RND)	*marA, H-NS, mdtC, baeR, acrA, acrB, CRP*	Chromosome

**Table 3:** Genomic characterization of putative virulence factors in the isolate H2730R*.

No.	Putative Virulence Factors	Genes	Organisms [Highest Homology]
1	Fimbrial adherence determinants	*csgA, csgB, csgC, csgD, csgE, csgF, csgG* *fimA, fimC, fimD, fimF, fimH, fimI, fimW,* *fimZ, lpfC, pegB, staB, staC, stcA, stcC,* *stgA, stgB, stkA, stkB, stkC, stkD, stkE and StkF*	*Salmonella enterica*
2	Nonfimbrial adherence determinants	*misL, ratB, shdA and sinH*	*Salmonella enterica*
3	Regulation	*phoP and phoQ*	*Salmonella typhimurium*
4	Toxin	*usp*	*Escherichia coli*
5	Motility	*flaA*	*Bordetella bronchiseptica*
6	Antiphagocytosis	*uge*	*Klebsiella pneumoniae*
7	Invasion	*ibeB*	*Escherichia coli*
8	Biofilm formation	*pgaC*	*Acinetobacter baumannii*

* The fimbrial adherence determinants consisted of the thin aggregative fimbriae (*csgA,* *csgB,* *csgC,* *csgD,* *csgE,* *csgF,* *csgG*), Type 1 fimbriae (*fimA, fimC, fimD, fimF, fimH, fimI, fimW, fimZ*), long polar fimbriae (*lpfC*), plasmid-encoded fimbriae (*pegB*), and other unique fimbriae (*staB, staC, stcA, stcC, stgA, stgB, stkA, stkB, stkC, stkD, stkE, and StkF*). The nonfimbrial adherence determinants included unique fimbriae (*misL, ratB, shdA,* and *sinH*). The regulation factor, toxin factor, and motility factor consisted of a two-component system (*phoP and phoQ*), colicin-like *usp*, and flagellin (*flaA*), respectively. The antiphagoctyosis, invasion, and biofilm formation determinants were composed of the capsule (*uge*), invasion of brain endothelial cell (*ibeB*), and biofilm-associated protein (*pgaC*), respectively.

## Appendix B

**Table A1:** Genomic features of Isolate H2730R.

Attribute	Value
Sequencing platform	Illumina MiSeq machine
Assembler	Skesa (version 2.3)
Assembly accession	VWTQ00000000
No. of Contigs	58
Genome size (bp)	5,299,408
DNA G + C (%)	51.8
Genome coverage (X)	99.0
Number of RNAs genes	70
Number of tRNAs genes	12
23S rRNAs	7
5S rRNAs	5
N50	518,368
L50	4
Number of subsystems	396
Number of CDSs	5135
Genes assigned to COGs	5006
Pseudo Genes	129

**Table A2:** A table showing the diversity of MLST (ST types) and allelic profiles of the seven housekeeping genes of all deposited *Citrobacter freundii* strains (n = 5) from South Africa.

Isolate	MLST	*arcA*	*aspC*	*clpX*	*dnaG*	*fadD*	*lysP*	*mdh*
944112508	ST156	7	15	63	54	13	5	12
944526466	NA	33	49	50	45	57	16	47
CF003	ST62	32	48	10	6	14	45	13
CF004	ST63	33	49	50	45	57	16	47
H2730R	ST498	5	16	14	54	103	5	15

NA, Not Available.
